# Effects of Individual Differences and Job Characteristics on the Psychological Health of Italian Nurses

**DOI:** 10.5964/ejop.v14i1.1478

**Published:** 2018-03-12

**Authors:** Maria Clelia Zurlo, Federica Vallone, Andrew P. Smith

**Affiliations:** a Department of Political Sciences, University of Naples Federico II, Naples, Italy; b Department of Humanistic Studies, University of Naples Federico II, Naples, Italy; c Centre for Occupational and Health Psychology, Cardiff University, Cardiff, Wales, United Kingdom; Department of Psychology, Webster University Geneva, Geneva, Switzerland; University of Wollongong, Wollongong, Australia

**Keywords:** stress in nursing, coping strategies, job characteristics, anxiety, depression, cross-cultural psychology

## Abstract

The Demand Resources and Individual Effects Model (DRIVE Model) is a transactional model that integrates Demands- Control-Support and Effort-Reward Imbalance models emphasising the role of individual (Coping Strategies; Overcommitment) and job characteristics (Job Demands, Social Support, Decision Latitude, Skill Discretion, Effort, Rewards) in the work-related stress process. The present study aimed to test the DRIVE Model in a sample of 450 Italian nurses and to compare findings with those of a study conducted in a sample of UK nurses. A questionnaire composed of Ways of Coping Checklist-Revised (WCCL-R); Job Content Questionnaire (JCQ); ERI Test; Hospital Anxiety and Depression Scale (HADS) was used. Data supported the application of the DRIVE Model to the Italian context, showing significant associations of the individual characteristics of Problem-focused, Seek Advice and Wishful Thinking coping strategies and the job characteristics of Job Demands, Skill Discretion, Decision Latitude, and Effort with perceived levels of Anxiety and Depression. Effort represented the best predictor for psychological health conditions among Italian nurses, and Social Support significantly moderated the effects of Job Demands on perceived levels of Anxiety. The comparison study showed significant differences in the risk profiles of Italian and UK nurses. Findings were discussed in order to define focused interventions to promote nurses’ wellbeing.

In recent years, the interest in the field of occupational stress has increased and has globally involved different work environments, working conditions, and type of employment. A growing body of research about health care workers’ wellbeing (Calnan, Wainwright, Forsythe, Wall, & Almond, 2001), and, in particular, a large number of studies conducted in many countries (Burke, Koyuncu, & Fiksenbaum, 2011; Drury, Craigie, Francis, Aoun, & Hegney, 2014; Hasselhorn, Tackenberg, & Peter, 2004; Kirkcaldy & Martin, 2000; Wu, Chi, Chen, Wang, & Jin, 2010) and in Italy (Lo Presti, 2013; Pino & Rossini, 2012; Pisanti, 2007; Pisanti, van der Doef, Maes, Lazzari, & Bertini, 2011; Pisanti et al., 2015; Taddei, Vieri, & Vanni, 2010; Viotti & Converso, 2016; Zurlo, Capasso, & Vallone, 2015) have recognised nursing as one of the professions subject to a higher level of stress. In particular, the shortage of staff and the consequent lack of nurses to deal with higher life expectancies and chronic illnesses, the issues related to a medically-dominated healthcare environment where nurses are still defining their role, and the lack of resources in a system expecting a high standard of excellence, should be considered together as common problems which nurses are dealing with (Greenglass & Burke, 2000; WHO, 2000, 2009).

Research applied different models to investigate work-related stress in nursing, reflecting the transition to transactional approaches to analyse occupational stress dimensions. Firstly, research on stress and health in nursing underlined in a transactional perspective the role played by coping strategies (Golbasi, Kelleci, & Dogan, 2008; Guido et al., 2009; Guido, Linch, Pitthan, & Umann, 2011; Healy & McKay, 2000; Lambert et al., 2004; Lu, Shiau, & Cooper, 1997; Pino & Rossini, 2012; Pisanti et al., 2015).

Transactional models of Demands-Control-Support (DCS; Karasek, 1979) and Effort-Reward Imbalance (ERI; Siegrist, 1996) have been the most widely used to analyse work-related stress in nursing, demonstrating, on the one side, that DCS dimensions were significantly related to job dissatisfaction, turnover intentions (Bourbonnais, Comeau, & Vézina, 1999; Gelsema et al., 2006), burnout and somatic complaints (Parkes, Mendham, & Von Rabenau, 1994), and, on the other side, that Effort-Reward Imbalance was significantly related to burnout (Hämmig, Braucheli, & Bauer, 2012; Hasselhorn, Tackenberg, & Peter, 2004; Schulz et al., 2009; Xie, Wang, & Chen, 2011), and psychological and physical disorders (Rotenberg, Silva-Costa, & Griep, 2014; Schreuder, Roelen, Koopmans, Moen, & Groothoff, 2010). Concerning Italian nurses, research referred to the DCS Model confirmed the important role played by high demands, low control and low support in predicting burnout and somatic complaints (Pisanti, 2007) and the application of the ERI Model highlighted significant associations of perceived levels of Effort and Rewards with Italian nurses’ psychological and physical health conditions (Zurlo, Capasso, & Vallone, 2015). However, despite the consensus about nursing as one of the most stressful occupations (Wheeler, 1998) and the attempts to unify the international policies developing the idea of transcultural nursing (Leininger & McFarland, 2006), countries’ specificities concerning health systems need to be acknowledged (Chambers et al., 2010; Schaufeli & Janczur, 1994). In particular, considering comparative studies on Italian and international healthcare contexts, re- search showed that the Italian health care service is characterized as having one of the highest rates of perceived lack of nursing staff (Pisanti et al., 2011), and higher levels of turnover intentions (Hasselhorn, Tackenberg, & Peter, 2004), as well as annual salary at levels below the European standards of the nursing profession (Arora et al., 2015).

Nursing research focused on the comparison between Italian and UK healthcare contexts has highlighted that Italian nurses reported significantly higher levels of disorganisation and lack of skill-set of staff than UK nurses (Glazer & Gyurak, 2008). Moreover, even with the significant progress made in this area, nursing is often considered as an auxiliary profession in Italy rather than in the UK, and the Italian nurses’ expertise is often not fully accredited and recognised in terms of skills and independence, creating ambiguous demands from physicians, patients and co-workers (Glazer & Gyurak, 2008; Prandstraller, 1995; Rocco, Cipolla, & Stievano, 2015).

## The DRIVE Model

In recent years, multi-dimensional approaches have been applied to evaluate occupational stress dimensions with a more complex perspective, and confirmed their validity to predict perceived levels of anxiety, depression as well as physical health conditions and sick leave in the nursing profession (Bourbonnais, Brisson, Vézina, Masse, & Blanchette, 2005; Gao et al., 2012a, 2012b; Griep, Rotenberg, Landsbergis, & Vasconcellos-Silva, 2011).

In this perspective, the Demands Resources and Individual Effects Model (DRIVE Model; Mark & Smith, 2008) embodies the new scientific directions, integrating DCS and ERI Models, giving particular emphasis to the role of individual dimensions (such as coping strategies) in influencing workers’ wellbeing, and displaying, above all the other multi-dimensional stress models, the major advantage of striking a good balance between simplicity and complexity (Mark & Smith, 2008).

The DRIVE Model has been successfully applied to evaluate occupational stress among various professional groups (Capasso, Zurlo, & Smith, 2016a, 2016b, 2018; Galvin & Smith, 2016; Mark & Smith, 2012a; Nelson & Smith, 2016), and, considering the nursing profession, the authors demonstrated the validity of the model (Mark & Smith, 2012b) highlighting significant associations between individual characteristics (i.e., Overcommitment and the coping strategies of Problem-focused, Seek Advice, Self-blame, and Escape/Avoidance) and job characteristics (i.e., Job Demands, Social Support, Skill Discretion, Effort and Rewards) in predicting perceived levels of Anxiety and Depression in a sample of 870 UK nurses. In particular, the risk model for Anxiety and Depression developed from a sample of UK nurses emphasised the negative role played by the individual characteristics of Self-blame coping strategy and Overcommitment (the latter being the best predictor for both Anxiety and Depression), as well as the protective role exercised by the individual characteristic of Problem-focused coping strategy and by the job characteristics of Rewards, Social Support and Skill Discretion. Moreover, in line with the rationale of the DRIVE Model as a transactional model, the authors carried out interaction analyses, demonstrating that Decision Latitude played a significant moderating role among UK nurses, buffering the effects of Job Demands on perceived levels of Anxiety. Nevertheless, despite the interest of the DRIVE Model in predicting effects of work-related stress on health conditions accounting for the complexity of the stress process through a multi-dimensional and transactional approach, it has been applied only in the UK context and no such attempt has been still made for its application in the Italian context.

## Aims

Considering the interest in the DRIVE Model theoretical framework (Mark, 2008; Mark & Smith, 2008) and in its application to the Italian context, the present study aims to test this model analysing main and interaction effects of individual characteristics (i.e., Coping Strategies, Overcommitment) and job characteristics (i.e., Effort, Rewards, Job Demands, Social Support, Skill Discretion, Decision Latitude) on perceived levels of Anxiety and Depression among Italian nurses. Moreover, considering that the DRIVE model, which has been developed in the UK, has not been previously tested in a sample of Italian nurses, and in order to adequately benefit from its application, the present study also aims at assessing, in a cross-cultural perspective, similarities and differences between Italian and UK nurses, comparing results from the present study with those emerged from the original application of the DRIVE Model in UK nurses (Mark & Smith, 2012b).

In line with the aims of the study, the following hypotheses have been tested:

*Hypothesis one*: the DRIVE Model dimensions of Coping Strategies (Problem-focused, Self-blame, Wishful Thinking, Seek Advice, Escape/Avoidance), DCS variables (Job Demands, Skill Discretion, Decision Latitude, Social Support), and ERI variables (Effort, Rewards, Overcommitment) have significant main and interaction effects on Italian nurses’ perceived levels of Anxiety and Depression, and Coping Strategies significantly add to the explained variance in outcomes, over and above the use of DCS and ERI Models.

*Hypothesis two*: There are significant differences between Italian and UK nurses with reference to the risk profiles defined by the DRIVE Model dimensions.

## Method

### Participants and Procedure

A cross-sectional study was conducted between May 2014 and June 2016 with the aims to test the DRIVE Model in the Italian context and to compare findings with those of a study conducted in a sample of UK nurses, applying the original DRIVE Model (Mark & Smith, 2008, 2012b). Chairmen of different public hospitals were contacted in order to achieve the authorization for submitting a questionnaire to the nursing staff. Nurses were then contacted directly asking them to complete the questionnaire. The respondents were informed of the objectives of the study and informed consent was obtained from all participants. Those who voluntarily enrolled in the study completed the questionnaire after a standardised oral introduction. Altogether, 450 out of 550 nurses (Men = 45.8%, *N* = 206; Women = 54.2%, *N* = 244) completed the questionnaire (response rate = 81.8%). Ages ranged from 20 to 65 years (*M* = 46.21, *SD* = 9.39). The study protocol was approved by the Ethical Committee of the university where this study took place, and the research was performed in accordance with the Declaration of Helsinki.

### Measures 

A questionnaire consisting of five sections was used:

1. An information section dealing with socio-demographic characteristics (i.e., sex; age).
2. The Ways of Coping Checklist-Revised (WCCL-R; Vitaliano, Russo, Carr, Maiuro, & Becker, 1985), which consists of 42 items on a 4-point Likert scale divided into five subscales: Problem-focused (Dealing with a stressful event using an optimistic and pragmatic attitude, e.g., “Made a plan and action and followed it”; Cronbach’s α = .88), Wishful Thinking (Wishes and fantasies of changing the negative situation/feelings, e.g., “Wished I could change the way that I felt”; Cronbach’s α = .85), Seek Advice (Talking to others and accepting their support and advice; e.g., “Talked to someone about how I was feeling”; Cronbach’s α = .75), Self-blame (Feeling responsible for the problem, e.g., “Criticized or lectured yourself”; Cronbach’s α = .78) and Escape/Avoidance (Behaving as if nothing happened, e.g., “Refused to believed it had happened”; Cronbach’s α = .74).
3. The Job Content Questionnaire (JCQ; Karasek et al., 1998), which consists of 27 items on a 4-point Likert scale divided into four subscales: Job Demands (e.g., “Do you have to work very fast?”; Cronbach’s α = .68), Social Support (e.g., “How often do you get help and support from your immediate superior?” Cronbach’s α = .85), Skill Discretion (e.g., “Do you have the possibility of learning new things through your work?”; Cronbach’s α = .68) and Decision Latitude (e.g., “Do you have a choice in deciding how you do your work?”; Cronbach’s α = .81). Skill Discretion and Decision Latitude subscales constituted the control dimensions.
4. The Effort-Reward Imbalance Questionnaire (ERI Test; Siegrist, 1996; Zurlo, Pes, & Siegrist, 2010), which consists of 23 items on a 5-point Likert scale divided into four subscales: Effort (e.g., “Over the past few years, my job has become more and more demanding”; Cronbach’s α = .79), Esteem Reward (e.g., “I received the respect I deserve from my superiors”; Cronbach’s α = .80), Material Reward (e.g., “Considering all my efforts and achievement, my salary/income is adequate”; Cronbach’s α = .84) and Overcommitment (e.g., “Work rarely lets me go, it is still on my mind when I go to bed”; Cronbach’s α = .76). Three subscales have been considered in the present study, and Esteem Reward and Material Reward were considered as one scale labelled as Rewards.
5. The Hospital Anxiety and Depression Scale (HADS; Zigmond & Snaith, 1983), which consists of 14 items on a 4-point Likert scale divided into two subscales: Anxiety (e.g., “Worrying thoughts go through my mind”; Cronbach’s α = .84) and Depression (e.g., “I have lost interest in my appearance”; Cronbach’s α = .78).

Scores were positively coded for each subscale and higher scores indicated the higher presence of that dimension. Anxiety and Depression scores were also converted into percentages and a score of 11 was considered the cut-off point in order to define the clinical cases (Zigmond & Snaith, 1983).

### Data Analysis

This study applied a cross-sectional design and the analyses carried out using SPSS (Version 20) were compared with findings from the UK application of the DRIVE Model among UK nurses.

In order to test the DRIVE Model among Italian nurses (*Hypothesis one*), firstly, a number of Descriptive Analyses of all the study variables were conducted, and clinical levels of Anxiety and Depression among Italian nurses were calculated (cut-off = 11; Zigmond & Snaith, 1983). Secondly, Pearson’s correlations were used to examine the associations between Coping Strategies (Problem-focused, Self-blame, Wishful Thinking, Seek Advice, Escape/Avoidance), DCS variables (Job Demands, Skill Discretion, Decision Latitude, Social Support), and ERI variables (Effort, Rewards, Overcommitment), and to explore their associations with perceived levels of Anxiety and Depression. Thirdly, Hierarchical Multiple Regression was carried out using the entry method to investigate the effects of individual and job characteristics in predicting perceived levels of Anxiety and Depression. The first block consisted of DCS variables, the second block consisted of DCS and ERI variables and, finally, Coping Strategies were included into the third block. The Variance Inflation Factor (VIF) and tolerance values were used for diagnosing multicollinearity, using VIF < 5 and tolerance > .40 as cut-off points (Allison, 1999; Belsley, Kuh, & Welsch, 1980). Sex and Age were also explored as independent variables included into the first block as control variables in order to consider their potential effects on model parameters. After Hierarchical Multiple Regression, a further set of Linear Regression Analyses were run to evaluate the interaction effects hypothesized between individual characteristics (Coping Strategies, Overcommitment) and Job Characteristics (DCS and ERI dimensions) in predicting perceived levels of Anxiety and Depression in Italian nurses (Cohen & Cohen, 1983; Cohen, Cohen, West, & Aiken, 2003).

In order to compare findings from Italian and UK studies (*Hypothesis two*), a comparison was drawn to evaluate statistically significant differences in Anxiety and Depression mean scores (*t*-test), in percentages of clinical levels of Anxiety and Depression (Chi-Square) and in Pearson’s correlations of individual and job characteristics with Anxiety and Depression between Italian and UK nurses (Fisher’ *r*-to-*Z*; Rosnow & Rosenthal, 2003).

## Results

### Testing the DRIVE Model Among Italian Nurses

Descriptive Statistics, Pearson’s correlations, Hierarchical Multiple Regression, and Linear Regressions Analyses were carried out to test the DRIVE Model among Italian nurses (*Hypothesis one)*. Descriptive statistics showed that Italian nurses’ mean Anxiety score was 6.12 (*SD* = 3.62) and the mean Depression score was 4.31 (*SD* = 3.18). Moreover, 19.3% of nurses showed clinical levels of Anxiety and 5.1% clinical levels of Depression.

Concerning the associations between individual characteristics (Coping Strategies, Overcommitment) and job characteristics (DCS and ERI dimensions), Pearson’s correlations highlighted that Problem-focused and Seek Advice coping strategies positively related to Skill Discretion, and that Self-blame, Wishful Thinking and Escape/Avoidance coping strategies negatively related to Skill Discretion and Decision Latitude. Moreover, perceived Social Support, Skill Discretion and Decision Latitude negatively related to perceived Job Demands and Effort and positively related to perceived Rewards (see Table 1).

**Table 1:** Descriptive Statistics and Correlations Between Coping Strategies, DCS and ERI Dimensions, Anxiety and Depression in Italian Nurses (N = 450)

Variable	*M*	*SD*	1	2	3	4	5	6	7	8	9	10	11	12	13	14
1. Problem-focused	24.58	8.16	-													
2. Self-blame	3.49	1.90	.37**	-												
3. Wishful Thinking	9.38	5.49	.27**	.62**	-											
4. Seek Advice	8.88	3.77	.67**	.43**	.43**	-										
5. Escape/Avoidance	9.27	5.35	.26**	.52**	.63**	.25**	-									
6. Overcommitment	11.77	3.52	.07	.22**	.23**	.08	.24**	-								
7. Effort	5.08	4.93	.08	.06	.17**	.06	.09*	.25**	-							
8. Rewards	19.47	4.08	.03	-.05	-.16**	.03	-.11*	-.26**	-.44**	-						
9. Job Demands	1.58	0.49	-.07	.01	.02	-.06	-.03	.02	.23**	-.26**	-					
10. Social Support	1.77	0.78	-.01	-.01	-.11*	-.01	-.14**	-.11*	-.19**	.33**	-.18**	-				
11. Skill Discretion	1.87	0.44	.13**	-.15**	-.16**	.11*	-.18**	-.03	-.11*	.20**	-.22**	.22**	-			
12. Decision Latitude	1.48	0.38	-.04	-.12**	-.26**	-.06	-.22**	-.10*	-.19**	.27**	-.23**	.22**	.26**	-		
13. Anxiety	6.08	3.62	-.07	.19**	.33**	.10*	.18**	.20**	.44**	-.31**	.20**	-.12**	-.22**	-.28*	-	
14. Depression	4.27	3.18	-.12**	.08	.21**	-.04	.18**	.10*	.30**	-.29**	.23**	-.20**	-.30**	-.25**	.59**	-

*p < .05. **p < .01.

Concerning the associations of the individual (Coping Strategies; Overcommitment) and job characteristics (DCS and ERI variables) with perceived levels of Anxiety and Depression, data revealed significant correlations in the expected directions (see Table 1).

Therefore, Hierarchical Multiple Regression was also carried out to validate the DRIVE Model in the Italian context by investigating the effects of the DRIVE Model’s individual and job characteristics in predicting perceived levels of Anxiety and Depression.

With respect to perceived levels of Anxiety, data from the last block including DCS variables, ERI variables and Coping Strategies showed that the individual characteristic of Problem-focused coping strategy and the job characteristic of Decision Latitude were significantly associated with low perceived levels of Anxiety, while the individual characteristics of Seek Advice and Wishful Thinking coping strategies and the job characteristics of Effort were significantly associated with high perceived levels of Anxiety (see Table 2). Moreover, with respect to perceived levels of Depression, the individual characteristic of Problem-focused coping strategy and the job characteristic of Skill Discretion were significantly associated with low perceived levels of Depression, while the job characteristics of Effort and Job Demands were significantly associated with high perceived levels of Depression (see Table 3). Data also revealed that among Italian nurses the ERI variable of Effort was the best predictor by standardized β weight for both Anxiety and Depression outcomes. The inclusion of Coping Strategies in the last block to test the validity of the whole DRIVE Model highlighted that it accounted for 35.1% of the variance in Anxiety (*R*^2^ = .351) with Delta *R*^2^ = .082 (see Table 2), and for 24.4% of the variance in Depression (*R*^2^ = .244) with Delta *R*^2^ = .039 (see Table 3). Conversely, the inclusion of Sex and Age as control variables didn’t reveal significant effects on all our findings. Finally, when checking for multicollinearity, the Variance Inflation Factors (VIFs) were < 5 and the tolerance values were > .40 for all the predictors, revealing the significant role of all the dimensions considered by the model.

**Table 2:** Hierarchical Multiple Regression: DCS, ERI and Coping Variables Against Anxiety (β Values)

Predictor variable	Step 1	Step 2	Step 3
Job Demands	.12*	.04	.04
Social Support	-.01	-.05	-.05
Skill Discretion	-.14**	-.13*	-.08
Decision Latitude	-.20***	-.14**	-.11*
Effort	-	.33***	.35***
Rewards	-	-.08	-.07
Overcommitment	-	.08	.04
Problem-focused	-	-	-.27***
Self-blame	-	-	.05
Wishful Thinking	-	-	.20**
Seek-Advice	-	-	.15*
Escape/Avoidance	-	-	-.01
*R*^2^	.14***	.27***	.35***
Delta *R*^2^	-	.13***	.08***

*p < .05. **p < .01. ***p < .001. Controlled by Sex and Age variables.

**Table 3:** Hierarchical Multiple Regression: DCS, ERI and Coping Variables Against Depression (β values)

Predictor variable	Step 1	Step 2	Step 3
Job Demands	.14*	.12*	.10*
Social Support	-.10*	-.03	-.04
Skill Discretion	-.21***	-.21***	-.16**
Decision Latitude	-.14*	-.10*	-.09
Effort	-	.19***	.19***
Rewards	-	-.10*	-.09
Overcommitment	-	.01	.01
Problem-focused	-	-	-.18*
Self-blame	-	-	-.01
Wishful Thinking	-	-	.09
Seek-Advice	-	-	.02
Escape/Avoidance	-	-	.09
*R^2^*	.14***	.20***	.24***
Delta *R^2^*	-	.06***	.04**

*p < .05. **p < .01. ***p < .001. Controlled by Sex and Age variables.

**Figure 1:**
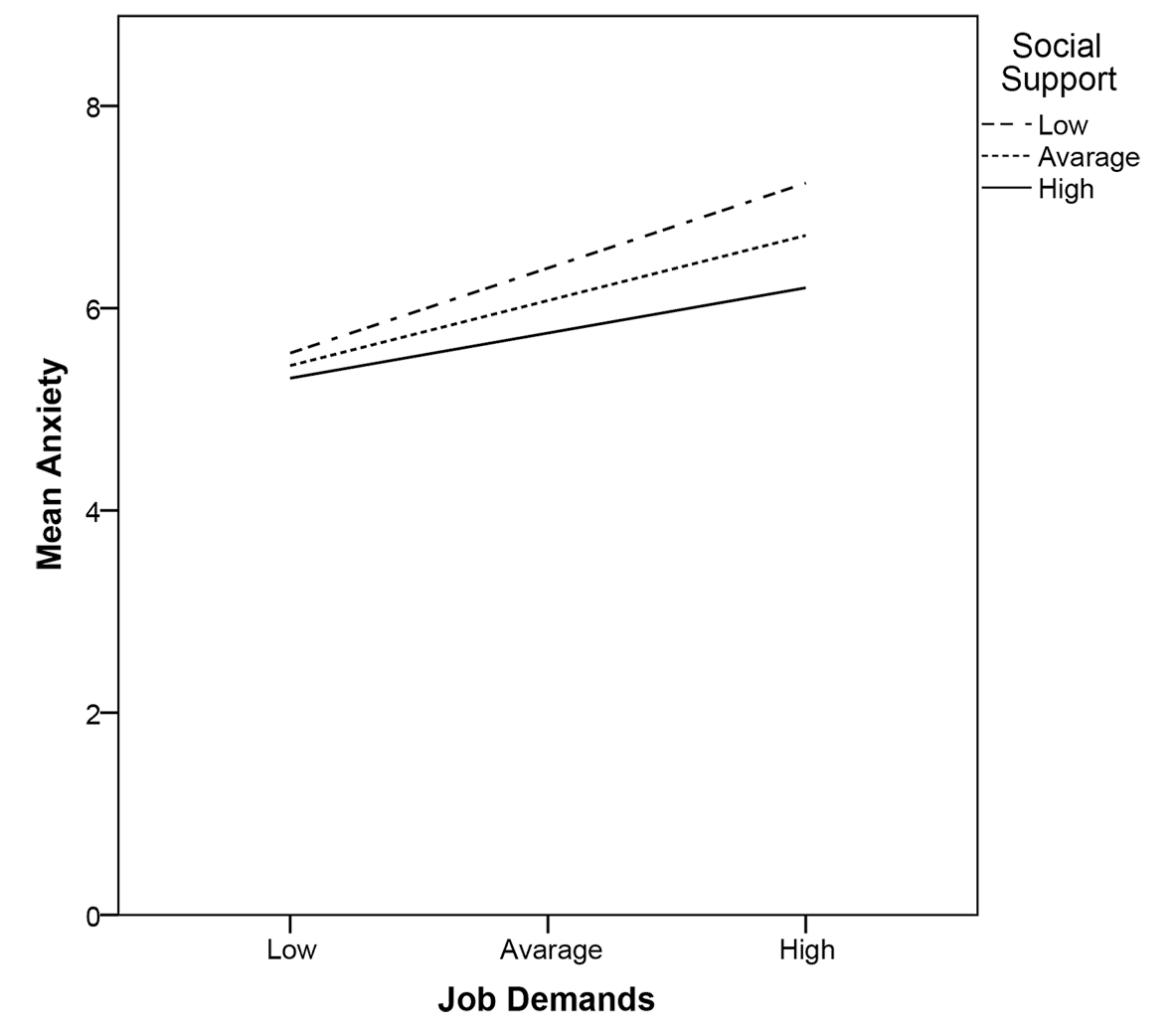
Interaction of Job Demands and Social Support in predicting Anxiety in Nurses.

The validation of the DRIVE Model was carried out also considering Linear Regression Analyses to test interaction effects, and data revealed a significant moderating effect of perceived Social Support in the relationship between Job Demands and Anxiety (Job Demands × Social Support against Anxiety: β = -.28; *p* = .044). Indeed, Figure 1 shows those individuals who perceived high levels of Job Demands were less anxious when they perceived more Social Support.

### Comparison of Italian and UK Nurses

*T*-test, Chi-Square Analyses and Fisher’ *r*-to-*Z* transformation analysis were used to compare findings from Italian and UK studies (*Hypothesis two*). With respect to the analysis of statistically significant differences in Anxiety and Depression mean scores (*t*-test), data revealed that Italian nurses reported significantly lower mean levels than UK nurses for both Anxiety (UK sample: *M* = 7.99, *SD* = 3.93; *t*(1318) = 8.410, *p* < .001) and Depression (UK sample: *M* = 4.86, *SD* = 3.23; *t*(1318) = 2.959, *p* = .003). Moreover, the comparison of the percentages of nurses reporting clinical levels of Anxiety and Depression (Chi-Square) showed that Italian nurses revealed a significantly lower percentage of perceived clinical levels of Anxiety than UK nurses (26.3%; χ2 (1, *N* = 1320) = 7.576, *p* = .005), while no significant differences emerged for clinical levels of Depression (5.1%; χ2 (1, *N* = 1320) = .296, *p* = .603).

Concerning the associations of DRIVE Model variables with perceived Anxiety and Depression among Italian and UK nurses, the analysis of Pearson’s correlations highlighted that Problem-focused coping strategy negatively related to Depression, while Wishful Thinking coping strategy, Escape/Avoidance coping strategy and Overcommitment positively related to Anxiety and Depression both in Italian and UK nurses. Moreover, Self-blame coping strategy positively related to Anxiety and Depression in UK nurses, while it related only to Anxiety in Italian nurses. Finally, Seek Advice coping strategy positively related to Anxiety only in Italian nurses. Concerning job characteristics considered in the DRIVE Model, all the variables investigated (Effort, Rewards, Job Demands, Social Support, Skill Discretion, and Decision Latitude) showed a significant correlation with Anxiety and Depression in both studies and in the expected directions. Moreover, with respect to the statistical comparison of the significant Pearson’s correlations (Fisher’ *r*-to-*Z* transformation analysis), data revealed in Italian nurses significantly smaller correlations of Escape/Avoidance coping strategy (Anxiety: *Z* = -2.96, *p* = .003; Depression: *Z* = -3.55, *p* < .001) and Overcommitment (Anxiety: *Z* = -7.64, *p* < .001; Depression: *Z* = -7.26, *p* < .001) with Anxiety and Depression, and of Self-blame coping strategy with Anxiety (*Z* = -5.68, *p* < .001). Furthermore, concerning job characteristics, significant smaller correlations emerged for Rewards (Anxiety: *Z* = 1.98, *p* = .047; Depression: *Z* = 2.77, *p* = .006) and Social Support (Anxiety: *Z* = 4.01, *p* < .001; Depression: *Z* = 3.79, *p* < .001) with Anxiety and Depression, and for Job Demands with Anxiety (*Z* = -2.41, *p* = .016) (see Table 4).

**Table 4:** Correlations of Individual and Job Characteristics With Perceived Levels of Anxiety and Depression in Italian and UK Nurses

	Italian sample (*N* = 450)		UK sample (*N* = 870)	
Characteristic	Anxiety	Depression	Anxiety	Depression
Problem-focused	-.07	-.12**	.04	-.10*
Self-blame	.19**	.08	.48**	.38**
Wishful Thinking	.33**	.21**	.34**	.28**
Seek Advice	.10*	-.04	.04	-.08
Escape/Avoidance	.18**	.18**	.34**	.37**
Overcommitment	.20**	.10*	.57**	.48**
Effort	.44**	.30**	.43**	.40**
Rewards	-.31**	-.29**	-.41**	-.43**
Job Demands	.20**	.23**	.33**	.26**
Social Support	-.12**	-.20**	-.34**	-.40**
Skill Discretion	-.22**	-.30**	-.21**	-.26**
Decision Latitude	-.28**	-.25**	-.21**	-.24**

*p < .05. **p < .01.

## Discussion and Conclusions

Findings showed that all the DRIVE Model dimensions had significant effects on Italian nurses perceived levels of Anxiety and Depression, confirming the validity of its application to the Italian context.

Firstly, data showed that 19.3% of nurses perceived clinical levels of Anxiety and 5.1% clinical levels of Depression, revealing significantly higher rates of psychological diseases than the Italian general population (i.e., 5.1% suffering from clinical Anxiety and 3% from Depression; de Girolamo et al., 2005, 2006; Lora, 2009). These data confirmed that nursing is a profession at high risk of work-related stress and psychological diseases, similarly to other care and educational professions (Zurlo & Pes, 2012; Zurlo, Pes, & Capasso, 2016).

Findings emerged from the Hierarchical Multiple Regression demonstrated the significant role of the recourse to Problem-focused coping strategies and of a higher perception of Decision Latitude and Skill Discretion in reducing the risk of developing anxiety and depressive symptoms. Conversely, data highlighted the negative effect of the recourse to Seek Advice and Wishful Thinking coping strategies on anxiety, as well as of perceived Job Demands on depression. Moreover, data emphasised the importance of acknowledging the negative role played by perceived Effort in the definition of Italian nurses’ risk profile, due to its higher weight in predicting psychological diseases among Italian nurses. Consequently, overall data gave specific indications to define both individual and organisational interventions, suggesting, on the one hand, to promote the use of problem solving coping strategies and to improve perceived control in terms of autonomy, decision latitude and variety of activities in nursing practice, and, on the one other hand, to reduce both the recourse to strategies focused on seeking advice and wishful thinking and the perception of high demands and efforts.

Furthermore, findings indicated perceived Social Support as a fundamental dimension, which played a significant moderating effect in the relationship between Job Demands and Anxiety, suggesting, above all, the importance of increasing the social support network within the workplace to effectively counteract the effect of perceived high demands on nurses’ psychological health conditions in the Italian context. In this perspective, the acknowledgement of the role played by individual characteristics, as well as the significant role played by perceived support in the Italian context, could be helpful to define interventions, particularly for jobs in which the reduction of demands is not easily achievable, such as nursing.

With respect to the comparative study (*Hypothesis two*), data confirmed the hypotheses of differences in the risk profiles of Italian and UK nurses. Firstly, data showed that Italian nurses reported significantly lower levels of both Anxiety and Depression than UK nurses and significantly lower percentages of clinical levels of Anxiety than UK nurses. Moreover, findings provided evidence for differences in the profiles of associations of the DRIVE Model’ dimensions with Anxiety and Depression between Italian and UK nurses, particularly considering individual characteristics (Coping Strategies and Overcommitment). If indeed, on the one hand, the protective role of Problem-focused strategy and the negative role of Wishful Thinking coping strategy were strongly supported in both studies, on the other hand, the important role played by the recourse to Self-blame and Escape/Avoidance coping strategies and the presence of the personality characteristic of Overcommitment in association with the higher risk for psychological health conditions were mainly emphasised in the UK study rather than in the present study. Conversely, the recourse to Seek Advice coping strategy represented a specific negative factor, which played a foremost role in determining Italian nurses’ risk to develop anxiety and depressive symptoms. Furthermore, considering job characteristics, all ERI and DCS dimensions were significantly related to Anxiety and Depression in both studies, but stronger evidence of negative effects of Job Demands and protective effects of perceived Rewards were provided in the UK study.

Generally, the comparison between Italian and UK nurses emphasised in particular the role played by different risk factors and resources. Concerning risk factors, the recourse to Seek Advice coping strategy emerged as the most significant individual risk factor among Italian nurses, while Self-blame and Escape/Avoidance coping strategies and Overcommitment were the most significant individual risk factors among UK nurses. Moreover, perceived levels of Effort were the most significant risk factor related to job characteristic among Italian nurses, as were Job Demands among UK nurses. Furthermore, concerning resources, perceived levels of Rewards represented protective factors more specifically indicated to define interventions among UK nurses. In this perspective, data supported the theoretical and practical value of the multi-dimensional approach of the DRIVE Model, which allows the definition of a more comprehensive risk profile, addressing a wide range of risk factors and resources.

In summary, data from both the testing of the DRIVE Model in the Italian context and the comparison between Italian and UK nurses provided theoretical and practical implications, allowing the identification of specific risk profiles referred to the articulation of ERI and DCS dimensions perceived in the work context with the individual coping strategies adopted by nurses. Therefore, the multi-dimensional and transactional perspective of the DRIVE Model should enable to define more complex and focused interventions both at individual and organisational levels to reduce perceived stress and to promote nurses’ wellbeing. In this perspective, with particular reference to the nursing profession in the Italian context, interventions at the individual level should be focused on reducing the recourse to coping strategies centred on Wishful Thinking and Seek Advice, less adequate to deal with physical and emotional sources of stress in nursing (e.g., pain, suffering and death; difficulties in the interpersonal relationships with physicians, patients and co-workers). Moreover, interventions should promote a more active way to deal with perceived work-related stress, increasing the use of coping strategies centred on individual competencies and pragmatic attitude. Interventions at the organisational level should aim to promote a more supportive social network and to improve role definition and decision latitude in the nursing profession.

Nevertheless, despite these results, some limitations need to be addressed. Firstly, the present study used a cross-sectional design, precluding any inference concerning the temporal associations between predictors investigated and outcomes, and no cause-effect relationships can be proposed. Secondly, two subscales from the Job Content Questionnaire revealed a low internal consistency, with Cronbach alphas below .70 (i.e., Job Demands Cronbach’s α = .68; Skill Discretion Cronbach’s α = .68). Thirdly, the total contribution of the considered variables to the explained variance was only acceptable, particularly with respect to Depression levels, and, considering also moderation effects, both Italian and UK studies supported only one significant interaction (i.e., respectively Job Demands × Social Support in predicting Anxiety in the Italian study and Job Demands × Decision Latitude in predicting Anxiety in the UK study), suggesting that the influence of other explanatory variables should be further explored.

All the limitations described above suggest the need for further research using the multi-dimensional perspective of the DRIVE Model, which has been developed to be a flexible framework, allowing also the addition of other significant variables that could be able to explain work-related stress process. In this perspective, the development of the DRIVE Model from its first application (Mark & Smith, 2008) has led to more enriched versions in different professions (Capasso, Zurlo, & Smith, 2016a, 2016b; Galvin & Smith, 2016; Nelson & Smith, 2016; Williams & Smith, 2016), and could be applied to further investigate occupational stress in nursing.
